# Association between air pollution and asthma admission among children in Hong Kong

**DOI:** 10.1111/j.1365-2222.2006.02555.x

**Published:** 2006-09

**Authors:** S L Lee, W H S Wong, Y L Lau

**Affiliations:** Department of Paediatrics and Adolescent Medicine, Queen Mary Hospital, The University of Hong Kong Hong Kong, China

**Keywords:** air pollution, asthma, children, Hong Kong, hospital admission

## Abstract

**Objective:**

To examine the association of air pollutants with hospital admission for childhood asthma in Hong Kong.

**Methods:**

Data on hospital admissions for asthma, influenza and total hospital admissions in children aged ≤18 years at all Hospital Authority hospitals during 1997–2002 were obtained. Data on daily mean concentrations of particles with aerodynamic diameter <10 μm (i. e. PM_10_) and <2.5 μm (i. e. PM_2.5_), nitrogen dioxide (NO_2_), sulphur dioxide (SO_2_), and ozone (O_3_) and data on meteorological variables were associated with asthma hospital admissions using Poisson's regression with generalized additive models for correction of yearly trend, temperature, humidity, day-of-week effect, holiday, influenza admissions and total hospital admission. The possibility of a lag effect of each pollutant and the interaction of different pollutants were also examined.

**Results:**

The association between asthma admission with change of NO_2_, PM_10_, PM_2.5_ and O_3_ levels remained significant after adjustment for multi-pollutants effect and confounding variables, with increase in asthma admission rate of 5.64% (3.21–8.14) at lag 3 for NO_2_, 3.67% (1.52–5.86) at lag 4 for PM_10_, 3.24% (0.93–5.60) at lag 4 for PM_2.5_ and 2.63% (0.64–4.67) at lag 2 for O_3_. Effect of SO_2_ was lost after adjustment.

**Conclusion:**

Ambient levels of PM_10_, PM_2.5_, NO_2_ and O_3_ are associated with childhood asthma hospital admission in Hong Kong.

## Introduction

There is substantial epidemiological evidence indicating a link between asthma morbidity including deterioration in lung functions, emergency department visits and hospital admission with outdoor air pollution levels [1]. Although it was generally believed that children are more vulnerable and susceptible than adults to air pollution exposure, [2] the PEACE project [3] which studied children aged 6–12 years in 28 regions of Europe, did not show any effects of particle matter, black smoke, sulphur dioxide (SO_2_) or nitrogen dioxide (NO_2_) on lung function, respiratory symptoms and bronchodilator use. The major limitation of the PEACE study has been the short observation period and it was suggested that increasing the length of the observation period, preferably including several seasons or years might provide better opportunities to tease out the relatively small effects of air pollution from the background noise in respiratory health indicators [4]. Hong Kong, one of the largest metropolitans in the world, is a good candidate for studying the health effects of air pollution with its dense population, detailed monitoring of air quality and hospital admissions. Since 1990s, there has been rapid decline in the air quality in Hong Kong attributed to vibrant economic activities in the region and nearby cities including Shenzhen and Guangzhou. In tune with the findings in PEACE, our previous prevalence study using International Study of Asthma and Related Allergies (ISAAC) methodology did not show a rise in prevalence of asthma in Hong Kong despite deteriorating air quality in past decades [5]. However, there was significant increase in prevalence of severe asthma symptoms including nocturnal awakenings by wheeze and nocturnal cough. We, therefore, performed a population-based time series analysis on daily hospital admission for asthma, a proxy for severe asthma attack in children less than 18 years old, with ambient air pollutants concentrations from 10 air quality-monitoring stations over Hong Kong. The result of the study may have important implication for health care policy in the region and may provide more evidences to support or dispute the acute effect of air pollutants in children with asthma.

## Methods

### Setting

Hong Kong is situated at the southeastern tip of China with a total area of 1102 km^2^ and a population of 6.816 million as of 2002. The population density is 6300 people/km^2^ and is one of the most densely populated cities in the world. Hong Kong's climate is sub-tropical, tending towards the temperate for nearly half the year. Temperatures can drop below 10°C in winter and exceed 31°C in summer. About 90% of the rainfall occurs between March and September. Air pollution derives mainly from motor vehicles, local power plants and industries in Guangdong because of the close proximity of these two regions.

### Hospital admission data

In Hong Kong, the Hospital Authority manages a total of 28 517 hospital beds and accounts for 90% of all hospital admissions. Since 1995, all Hospital Authority inpatient data, including demographic characteristics, dates of admission and discharge, diagnoses and procedures on discharge using the International Classification of Diseases, 9th, Revision, Clinical Modification (ICD-9-CM), have been stored in a central-computerized database. We obtained data on daily hospital admissions for asthma (ICD-9-CM code 493) as primary diagnosis upon discharge, influenza (ICD-9-CM code 487) as primary diagnosis upon discharge for control of viral respiratory seasonal epidemics and the total hospital admissions in patients ≤18 years of age from all hospital authority hospitals from January 1997 to December 2002.

### Data on air quality and weather

**Fig. 1:**
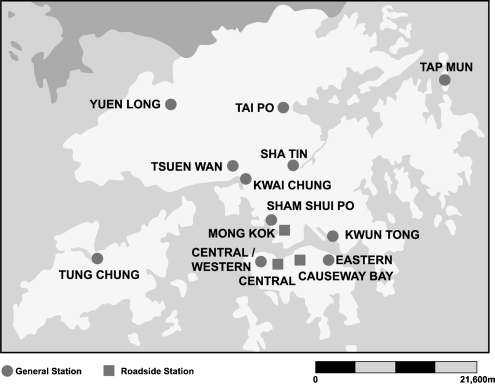
Location of the stations for monitoring air quality.

Data on five outdoor air pollutants including SO_2_, NO_2_, respirable suspended particulates (RSP) understood as particulate matter 10 and 2.5 μm in aerodynamic diameter (PM_10_ and PM_2.5_), respectively, and also ozone (O_3_) were obtained from the Environmental Protection Department, Hong Kong SAR [6]. There were nine stations for monitoring general air quality and it was increased to 11 stations in 2000 Further there are stations for roadside air quality across the territory. All stations are situated close, to residential areas, except the one in Tap Mum (Fig. 1), the data of which was excluded. The hourly concentration record of each air pollutant from each included station was retrieved and the daily mean of each air pollutant was calculated. Then the arithmetic mean of each air pollutant from all included stations was calculated. In each station, for O_3_ the 8-hourly (9:00–17:00 hours when O_3_ concentrations were highest) means were taken as daily data, if there were at least six valid hourly data each day, and for the other pollutants the 24-hourly means were used as daily data if there were at least 18 valid hourly data. When daily record of individual air pollutant from a particular station was considered to be invalid, the daily mean of that air pollutant was not included in mean calculation. In general, the concentration of gaseous pollutants and RSP are determined continuously by automatic analyzers. Manually operated high volume samplers using the gravimetric methods are also used regularly to measure the RSP. Meteorological data including mean temperature, humidity and atmospheric pressure were obtained from the Hong Kong observatory [7].

### Statistical analysis

Pearson's correlation was used to determine the correlation between stations for daily concentrations of each air pollutant and so for correlation between daily concentrations of air pollutants and meteorological variables.

Semi-parametric Poisson's regression with generalized additive models for adjustment of over-dispersion using SAS version 8.02 was used to model the daily counts of asthma admissions. The core model included smooth function of the day of study, spline smooth functions of mean daily temperature and relative humidity, daily hospital admissions for influenza and total daily hospital admission and indicator variables for day of the week and holidays to avoid over control for the effect of the pollutants and to account for the over-dispersion for the variable and possible population changes during the study period.

Before adding the air pollutant variables into the model, the effects of temperature and humidity on day of admission and up to 5 days before admission (i.e. at lag 0, 1, 2, 3, 4 and 5) were investigated and modelled using minimization of Akaike's Information Criteria (AIC) and lack of over- or under-fitting in the residual correction for autocorrelation. Both mean daily temperature and relative humidity on day of admission (at lag 0) were selected in the subsequent model as they had the best-combined fit. After building up the core regression model for temperature and humidity-related hospital admissions, single pollutant was entered into the regression, and the effects of the pollutant on the day of admission and the previous 5 days (i.e. at lag 0, 1, 2, 3, 4 and 5) were examined to account for potential delays in disease incidence after important exposures. Multi-pollutant models were run for pollutants that were significant in the single pollutant analysis, and the lag that had the strongest univariate effect was tested. The result was expressed as per cent increase with 95% confidence intervals (95% CI) in daily admission with each increment of an inter-quartile range (IQR) change of each pollutant.

## Results

**Fig. 2:**
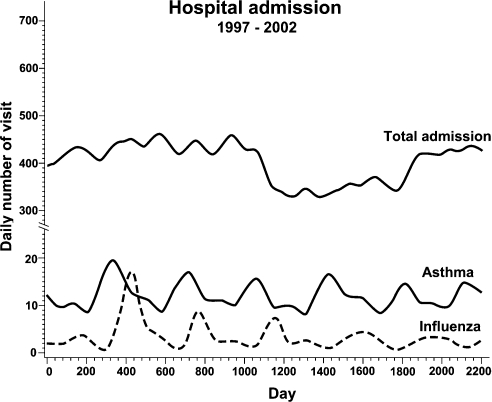
Spline smooth of total hospital admission, asthma admission and influenza admission from years 1997 to 2002.

**Fig. 3:**
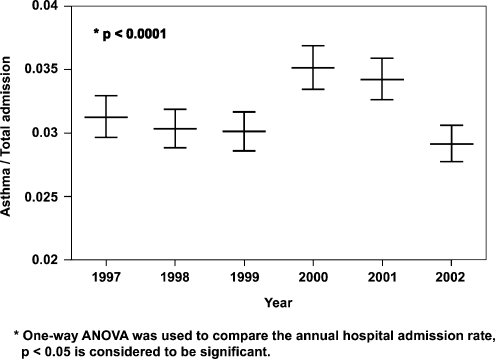
Mean ratio with 95% confidence interval (95% CI) of asthma admission to total hospital admission from 1997 to 2002.

Table 1 summarizes data for hospital admissions, and for meteorological and pollution variables. There were 879 384 total hospital admissions, 26 663 asthma admissions and 5821 influenza admissions for children ≤18 years of age recorded with the respective daily average admission of 401, 12.1 and 2.7 over the 6-year study period. Standardized annual total hospital admission, admission for asthma and influenza were about 999, 31 and 7 per 10 000 children ≤18 years, respectively, based on population figures [8]. There was a decreasing trend in annual total hospital admission until year 2002, while annual asthma admission rate remained fairly constant over these 6 years (Fig. 2), resulting in an increasing contribution of asthma admission to total hospital admission (*P* < 0.001) (Fig. 3).

**Table 1:** Summary of environmental variables and daily asthma hospital admission data in Hong Kong, 1997–2002

		Percentiles		
		
Pollutant variable	Mean±SD	25th	50th	75th	IQR
Environmental variables					
Temperature (°C)	23.7 ± 4.8	20.0	24.8	27.8	7.8
Humidity (%)	78.1 ± 10.0	74	79	85	11
Rainfall mm	7.4 ± 23.5	0	0	2.3	2.3
Pollutant variable (μg/m^3^)					
PM_10_ (24 h)	56.1 ± 24.2	37.3	51.1	70.7	33.4
PM_2.5_ (24 h)	45.3 ± 16.2	33.4	43.0	54.0	20.6
SO_2_ (24 h)	17.7 ± 10.7	10.6	15.2	21.7	11.1
NO_2_ (24 h)	64.7 ± 20.9	49.7	63.5	76.8	27.1
O_3_ (8 h)	28.6 ± 16.0	15.9	25.4	38.9	23
Hospital daily admission (number)	401 ± 90.7	332	404	468	136
Asthma daily hospital admission (number)	12.1 ± 5.4	8	11	15	7
Influenza daily hospital admission (number)	2.7 ± 4.3	0	1	3	3

Observation period was 2191 days, total, hospital admission was 879 384, asthma admission was 26 663 and influenza admission was 5821.

PM_10_, aerodynamic diameter <10 μm; PM_2.5_, aerodynamic diameter <2.5 μm; SO_2_, sulphur dioxide; NO_2_, nitrogen dioxide; O_3_, ozone.

Table 2 shows the correlations among the air pollution and weather variables. There was a marked seasonal variation in ambient concentrations of PM_10_, NO_2_ and O_3_, all of which were lowest in warm season (April–September) and highest in cool season (October–March). In contrast, there is no significant seasonal fluctuation of SO_2_. Strong correlations were observed between PM_10_ and PM_2.5_ (*r* = 0.90), NO_2_ (*r* = 0.78), O_3_ (*r* = 0.48) and SO_2_ (*r* = 0.37); between SO_2_ and NO_2_ (*r* = 0.49), and between NO_2_ and O_3_ (*r* = 0.35). The correlation of PM_2.5_ to other pollutants was similar to the correlation of PM_10_ with these pollutants The pattern of correlations remained similar across seasons. About 70% of PM_10_ is in the fraction of PM_2.5_, and the main source is motor vehicle exhaust in Hong Kong [9]. The between-station correlations were high for all air pollutants with mean (range) 0.93 (0.85–0.97) for PM_10_, 0.78 (0.53–0.94) for NO_2_, 0.82 (0.57–0.93) and 0.61 (0.53–0.94) for SO_2_.

**Table 2:** Pearson's correlation coefficients among environmental variables, Hong Kong 1997–2002

	Temperature	Humidity	Rainfall	PM_10_	PM_2.5_	SO_2_	NO_2_
*Whole year*							
Humidity (%)	0.22						
Rainfall (mm)	0.12	0.35					
PM_10_ (μg/m^3^)	−0.33	−0.48	−0.27				
PM_2.5_ (μg/m^3^)	−0.21	−0.35	−0.24	0.90			
SO_2_ (μg/m^3^)	0.13	−0.15	−0.09	0.37	0.47		
NO_2_ (μg/m^3^)	−0.38	−0.37	−0.20	0.78	0.75	0.49	
O_3_ (μg/m^3^)	−0.09	−0.40	−0.20	0.48	0.36	−0.17	0.35
*Cold season (October–March)*							
Humidity (%)	0.15						
Rainfall (mm)	0.03	0.27					
PM_10_ (μg/m^3^)	0.01	−0.44	−0.24				
PM_2.5_ (μg/m^3^)	0.05	−0.29	−0.21	0.90			
SO_2_ (μg/m^3^)	0.01	−0.18	−0.12	0.53	0.59		
NO_2_ (μg/m^3^)	0.00	−0.33	−0.18	0.69	0.70	0.69	
O_3_ (μg/m^3^)	0.27	−0.36	−0.15	0.33	0.21	−0.24	0.08
*Warm season (April–September)*							
Humidity (%)	−0.32						
Rainfall (mm)	−0.16	0.46					
PM_10_ (μg/m^3^)	−0.18	−0.38	−0.23				
PM_2.5_ (μg/m^3^)	−0.03	−0.28	−0.21	0.89			
SO_2_ (μg/m^3^)	0.23	−0.21	−0.13	0.39	0.48		
NO_2_ (μg/m^3^)	−0.31	−0.25	−0.12	0.80	0.74	0.51	
O_3_ (μg/m^3^)	−0.30	−0.43	−0.20	0.60	0.47	−0.11	0.52

PM_10_, aerodynamic diameter <10 μm; PM_2.5_, aerodynamic diameter <2.5 μm; SO_2_, sulphur dioxide; NO_2_, nitrogen dioxide; O_3_, ozone.

**Fig. 4:**
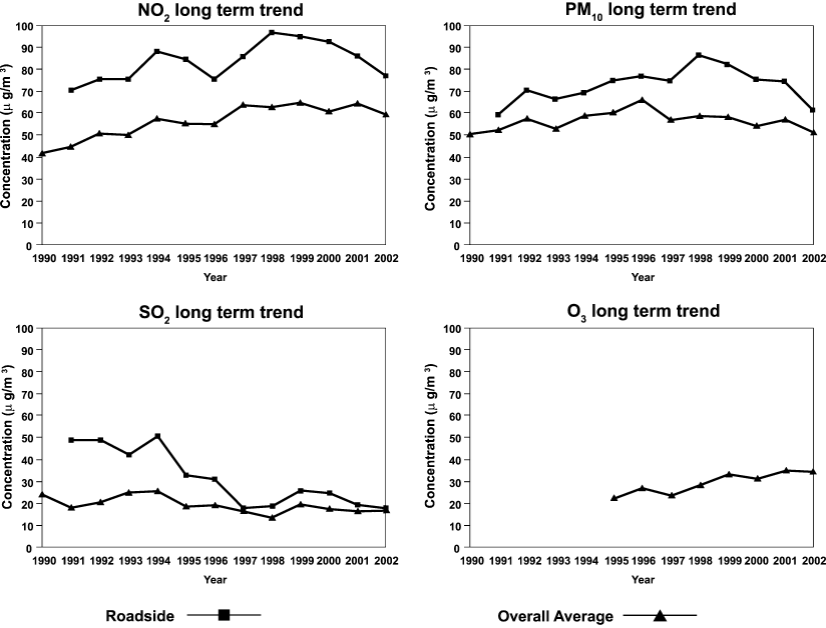
Long-term trends of four pollutants studied.

There has been a noticeable change in ambient decline in air quality in Hong Kong since 1990s (Fig. 4). SO_2_ concentration has decreased significantly after the introduction of fuel quality restriction in 1990. The 1997 annual averages were about 40–64% lower than that before enforcement of fuel restriction. The roadside concentration dropped to 18 μg/m^3^ in 2002. The overall NO_2_ level was over 60 μg/m^3^, while the roadside NO_2_ level persistently exceeded the permissible limit of 80 μg/m^3^ over all these years. Although there was a decreasing trend of (RSP) over the study period, the roadside RSP trend remained well above the permissible limit of 55 μg/m^3^. The recognition of the steep rise in the overall O_3_ average in the past few years (76.5% from 1997 to 1999) was enabled by the addition of seven stations in O_3_ monitoring. Nevertheless, data from old stations, which have been monitoring O_3_ since 1990 also revealed a slow but steady rising trend in the last decade.

Table 3 summarizes the results of the single-pollutant analysis for asthma admission up to 5 lag days. All the five pollutants studied were associated with an increase in daily asthma admission. An increase in IQR of daily mean concentration of PM_10_ (33.4 μg/m^3^), PM_2.5_ (20.6 μg/m^3^) and NO_2_ (27.1 μg/m^3^) were associated with increase in daily asthma admission on the day of admission and all the 5 days before admission (lags 1–5). The most significant increase in asthma admission was 9.08% (95% CI 7.26–10.93) per IQR change of NO_2_ level 3 days before admission (lag 3). The corresponding increase in asthma admission were 7.45% (5.58–9.35) per IQR change of PM_10_ on lag 4, 6.59% (4.51–8.72) per IQR change of PM_2.5_ on lag 4, 5.97% (4.10–7.89) per IQR change of O_3_ (23.0 μg/m^3^) on lag 3 and 1.46% (0.19–2.74) per IQR change of SO_2_ (11.1 μg/m^3^) on lag 5.

**Table 3:** Percentage increase in daily hospital admissions for asthma for age <=18 year old per interquartile range (IQR) increase of pollutants at different lags, Hong Kong 1997–2002

Lag	PM_10_; IQR: 33.4 μg/m^3^	PM_2.5_; IQR: 20.6 μg/m^3^	SO_2_; IQR: 11.1 μg/m^3^	NO_2_; IQR: 27.1 μg/m^3^	O_3_; IQR: 23.0 μg/m^3^
0	4.97 (2.96–7.03)	5.10 (2.95–7.30)	−1.57 (−2.87–−0.26)	4.37 (2.51–6.27)	2.34 (0.40–4.31)
1	5.71 (3.78–7.68)	5.00 (2.88–7.16)	−1.77 (−3.06–−0.46)	5.88 (4.00–7.70)	4.59 (2.71–6.51)
2	6.40 (4.51–8.32)	4.83 (2.75–6.95)	−1.15 (−2.42–0.14)	7.19 (5.37–9.04)	5.97 (4.10–7.89)*
3	7.25 (5.38–9.16)	4.83 (2.78–6.93)	0.82 (−0.45–2.11)	9.08 (7.26–10.93)*	3.87 (2.02–5.75)
4	7.45 (5.58–9.35)*	6.59 (4.51–8.72)*	1.40 (0.13–2.69)	7.64 (5.84–9.48)	2.41 (0.58–4.26)
5	5.96 (4.11–7.85)	5.24 (3.18–7.34)	1.46 (0.19–2.74)*	6.40 (4.60–8.22)	0.86 (−0.94–2.7)

Data presented as percentage increase (95% confidence interval). PM_10_: aerodynamic diameter <10 μm; PM_2.5_,aerodynamic diameter <2.5 μm; SO_2_, sulphur dioxide; NO_2_, nitrogen dioxide; O_3_, ozone.

* Highest percentage change after control for the mean daily temperature, relative humidity, daily hospital admissions for influenza, total daily hospital admission and indicator variables for day of the week and holidays.

Table 4 shows that association between asthma admission with change of NO_2_, PM_10_, PM_2.5_ and O_3_ levels remained significant after adjustment for multi-pollutants effect and confounding variables, with increase in asthma admission rate of 5.64% (3.21–8.14) at lag 3 for NO_2_, 3.67% (1.52–5.86) at lag 4 for PM_10_, 3.24% (0.93–5.60) at lag 4 for PM_2.5_ and 3.76% (0.47–6.26) at lag 2 for O_3_. Effect of SO_2_ was lost after adjustment. Owing to the multi-collinearity of PM_10_ and PM_2.5_, they were separately put into the multi-pollutants model. For the other pollutants, the result is similar when putting either PM_10_ or PM_2.5_, and only the set of results when PM_10_ was included in the multi-pollutants model is presented.

**Table 4:** Percentage increase in daily asthma hospital admission per inter-quartile range (IQR) increase of pollutants in single- and multi-pollutant models, Hong Kong 1997–2002

Variable	Lag	Single-pollutant model*	Multi-pollutant model†
PM_10_	4	7.45 (5.58–9.35)	3.67 (1.52–5.86)‡
PM_2.5_	4	6.59 (4.51–8.72)	3.24 (0.93–5.60)‡
SO_2_	5	1.46 (0.19–2.74)	0.81 (−0.75–2.4)
NO_2_	3	9.08 (7.26–10.93)	5.64 (3.21–8.14)
O_3_	2	5.97 (4.10–7.89)	3.76 (0.47–6.26)

Data presented as percentage increase (95% confidence interval). PM_10_, aerodynamic diameter <10 μm; PM_2.5_, aerodynamic diameter <2.5 μm; SO_2_, sulphur dioxide; NO_2_, nitrogen dioxide; O_3_, ozone.

* Single pollutant model results for the most significant single lag day.

† Estimates from regression models containing five pollutants simultaneously.

‡ Owing to the multi-collinearity of PM_10_ and PM_2.5_, they are separately put into the multi-pollutant model.

## Discussion

Our study showed that ambient level of both particulates (PM_10_, and PM_2.5_) and gaseous pollutants (NO_2_ and O_3_) are associated with childhood asthma hospital admission in Hong Kong. The effects of these four pollutants were independent as the associations remained significant after adjustment in the multi-pollutant model. The validity of our study is supported by long study period, reliable central-computerized source of hospital admission data for over 90% of the population and good air-quality monitoring system of international standard.

There have been much debate on the appropriate statistical methods in analysing the effect of air pollution with different heath outcomes and comparing the results across different regions. There are several important approaches, e.g. the National Mortality and Morbidity Air Pollution Study (NMMAPS) in the United States focused on the 20 largest cities during 1987–1994 [10, 11], and Air Pollution and Health: a European Approach (APHEA) 1 and 2 in European countries [10, 12]. It was later discovered that there was a problem in statistical model of the NMMAPS [13], which led to an overestimation of the effect reported although qualitative conclusions did not change. APHEA 1 attempted to standardize the statistical method for comparison across different European cities using Poisson's time series models and combine the city-specific estimates of the effect of air pollution using meta-analysis. Parametric approach in modelling seasonality and weather was used, but it was subjected to potential biases [14]. Nevertheless, non-parametric smoothing function, however, does not perform well when there are a large number of independent variables in the model and this could be one of the reasons for lacking significant association between ambient air pollutants and asthma hospital admission in some of the previous studies. We adopted the generalized additive model in our data analysis which is similar to that of APHEA 2 [15]. It allows the adjustment of both parametric and non-parametric data in the same model. We included several important confounders in our model. Susceptibility to epidemics of respiratory infections and the effects of school holidays are important considerations for asthma admission in children. The increase in traffic across the border between Hong Kong and mainland China may lead to a fluctuation in population size during holidays. Birth rate has also been dropping in Hong Kong over the past decade. Thus, in addition to adjustment for day of the study, day of the week and calendar holidays, total hospital admissions for children ≤18 years old were also considered as a proxy for the population denominator. Our recent study showed that influenza is an important cause of hospitalization among children in Hong Kong, with rates exceeding those reported for temperate regions [16] and have influenza epidemic has been included in our model. We did not take exposure of pollens into account as suggested by other studies [17, 18] because pollenosis is uncommon and there is no official data available in our locality.

Information bias related to accuracy and completeness of data on hospital admissions for asthma could account for inconsistencies of the result among different studies or across different age groups even within the same study. We obtained data from a central computerized database. The computerized system was launched in all hospitals under the Hospital Authority in Hong Kong since 1995 and the data became complete and reliable since 1997. We restricted our study population to all subjects younger than 18 years old. All patients under this age have been managed in paediatric wards in Hong Kong since 1997, a change in practice that has been adopted in many other developed countries. The difference between vulnerability and susceptibility of children and adult to air pollution exposure has been reviewed in detail [2]. Moreover, the relative lower contribution of asthma admission to total admission for respiratory diseases in adults and elderly would render less power to detect the associations of asthma admission with air pollutants when the studies were carried out for whole age group with same period of time [15, 19]. A recent study from Atlanta, USA [20] showed that the association for paediatric asthma emergency visits in relation to PM_10_ was stronger than those for adult asthma visits. We attempted to include the longest possible duration of observation period but this could only be extended up to year 2002 as the hospital admission for children was greatly affected by the SARS in 2003. A post-priori calculation of our sample size of 2191 days of observation showed that it could detect a low correlation rate of *r* = 0.06 (95% CI 0.02–0.10) between air pollutants and asthma admission at 80% power and 5% of α.

Biological plausibility of respirable particulates on respiratory morbidity was reviewed [21]. However, there were few studies actually showing significant association of respirable particulates with asthma admission, particularly in children (Table 5). Some of these studies [17,23–28] showed significant association of asthma admission in children with ambient NO_2_ level but none except early Ontario study [23] showed significant association with particulate matter. Particulate matter was considered as a surrogate for other pollutants with no contribution of its own [29]. The apparent lack of effect of the studies could be attributed to the use of black smokes [17, 24–26] and PM_13_ [27] instead of PM_10_ in their study. Using a newer statistical model and use of data on PM_10_, APHEA 2 was able to show that PM_10_ was positively associated with increased number of admissions for asthma in children aged 0–14 years [15]. Nevertheless, there was substantial heterogeneity in results among the participating cities and the association between asthma admissions and PM_10_ was lost after inclusion of NO_2_. Our study used a model similar to that of APHEA 2 but we also adjusted for the population change using total hospital admission as a proxy and included admissions of children aged above 14 to below 18 years old. This would provide a more complete and reliable estimation of both numerators and denominators in calculating the possible association. Our study showed a strong association of PM_10_ and PM_2.5_ with asthma admission and the effect remained significant after adjustment in the multi-pollutants model. This argued against particulate matter as a surrogate only. A recent systematic review of panel studies showed an adverse effect of particulate air pollution that is greater for PM_2.5_ than for PM_10_ on lung function and symptoms of children with asthma [30]. However, there have not been any panel studies or time series studies of the effect of PM_2.5_ on hospital admission for asthma in children. To the best of our knowledge, our study is the first to show such a relationship.

**Table 5:** Time series population based studies* of short term effect of air pollutants on asthma hospital admission in children

Author	Year	Place	Months	Age	NO_2_	SO_2_	Particles	O_3_	Remarks	Statis method
Bates [22]	74, 76–83	Ontario	1,2,7,8	0–14	Sign (winter)	NS	Sign	NS	Change in ICD code in 1979	Pearson correlation
Burnett et al. [23]	83–88	Ontario	1–12	2–34	NA	Sign	NA	Sign	Only two pollutants measured	Pearson correlation
Sunyer et al. [24]	86–92	Helsinki	1–12	0–14	NS	NS	NS (BS)	NS		APHEA1
	London	1–12	0–14	Sign	Sign	NS (BS)	NS		
	Paris	1–12	0–14	NS	Sign	NS (BS)	NS		
Anderson et al. [17]	87–92	London	1–12	0–14	Sign	Sign	NS (BS)	NS		APHEA1
Morgan et al. [25]	90–94	Sydney	1–12	1–14	Sign	NS	NS (BSP)	NS		Similar to APHEA 1
Petroeschevsky et al. [26]	87–94	Brisbane	1–12	0–14	Sign	NS	Sign neg (BSP)	Sign	Sign assoc	APHEA 1
									Between GI disease	
								And SO2	
Fusco et al. [27]	95–97	Rome	1–12	0–14	Sign	NS	NS (PM13)	NS	Sign assoc with CO	Similar to APHEA 2
Atkinson et al. [15]	94–96	Barcelona	1–12	0–14	NA	NA	NS (PM10)	NA		APHEA 2
92–94	Birmingham					Sign (PM10)			
92–94	London					NS (PM10)			
90–97	Milan					Sign (TSP)			
92–95	The Netherlands					NS (PM10)			
92–96	Paris					NS (PM13)			
95–97	Rome					NS (TSP)			
94–96	Stockholm					NS (PM10)			
	Summary					Sign (PM10)†			
Barnett et al. [28]	98–01	Brisbane	1–12	0–14	Sign	NS	Sign (PM10)‡	NS		Cross-over study
	Christchurch								
	Melbourne								
	Perth								
	Syndey								

NO_2_, nitrogen dioxide; O_3_, Ozone; SO_2_, sulphur dioxide; CO, carbon monoxide; TSP: total suspended particles; BS, black smoke; BSP, particles measured by nephelometry (air heated to 70°C to control for humidity and the light scattering of dry particles is measured); PM_10_, PM_13_, particles with an aerodynamic diameter of less than 10 and 13 μm; Sign, significant; NS, not significant; NA, data not available.

* Only studies with separate result in children were included for comparison.

† Pooled estimates in single pollutant model; effect lost after inclusion of NO_2_, SO_2_, CO but not O_3_ in two pollutants model.

‡ Effect lost after matching on NO_2_.

NO_2_ has been shown to activate oxidant pathways, enhance airway responses to inhaled allergens in asthmatic individuals and impair function of alveolar macrophages and epithelial cells. Exposure to NO_2_ occurs both indoors from gas cooking appliances and outdoors, mainly from vehicle exhausts. Epidemiological studies showed conflicting results of the effect of NO_2_ with some showed an association between indoor exposure and respiratory symptoms in children whereas other failed to confirm it. Living close to main road is shown to be a risk factor for wheezing illness in children [31]. Another well-designed prospective study from Southampton showed that high personal exposure to NO_2_, at levels within air quality standard in the week before the start of a respiratory viral infection is associated with asthma exacerbation in children [32]. In Hong Kong, the roadside NO_2_ and RSP level has persistently exceeded the permissible limits in the past 10 years which could be attributed to the incessant increase in road traffic [8] and trapping of polluted air including vehicle exhausts by the city's tall buildings, the so-called canyon effect. Many residential areas are situated in close proximity to busy road traffic. As outdoor particles readily penetrate indoors, we believed that PM measured at outdoor fixed sites would correlate closely with personal exposure. In addition, exposure misclassification from using central regional PM data should have biased towards the null findings. A recent panel study in children with asthma in fact showed that ambient-generated component of PM_2.5_ exposure has stronger association than indoor-generated component with increase in exhaled nitric oxide, a marker of airway inflammation [33]. All these factors helped to explain the relative strong association of particulate matter and NO_2_ with hospital admission for asthma in our study when compared with studies from other cities.

Exposure to O_3_ can increase respiratory symptoms and lead to acute hospital admissions for asthma. Ambient O_3_ is formed by a series of complicated photochemical reactions of oxygen, nitrogen oxides and volatile organic compounds under sunlight. Nitric oxide emissions from motor vehicles have scavenging effect on O_3_ and areas with heavy traffic flow normally have lower O_3_ levels. This is reflected in ambient O_3_ level of Hong Kong where much higher O_3_ level is detected in rural than urban stations. O_3_ provocation studies showed that effective dose depends on concentration, duration of exposure and degree of exercise. The close proximity of most residential areas to heavy road traffic and the relative lack of outdoor activities in children in Hong Kong might explain the less marked effect of O_3_ on asthma admission when compared with that of PM_10_ and NO_2_.

The restrictions on sulphur content of fuel in Hong Kong in July 1990 led to immediate improvement in air quality and immediate and long-term beneficial health effects [34–36]. Absence of association of SO_2_ with asthma admission was attributed to low ambient SO_2_ level over these years. Our study added evidence of health benefits related to this industrial fuel intervention.

In conclusion, our study showed that ambient levels of PM_10_, PM_2.5_, NO_2_ and O_3_ in our locality were associated with childhood asthma hospital admission in Hong Kong. We speculate that the increasing prevalence of severe asthma symptoms in our previous ISAAC study [5] and the rising contribution of asthma admission to total hospital admission in children over the study period could be related to the worsening air pollution in our population. This called for the attention of health policy makers towards the ever-rising air pollutants level in Hong Kong. Our study also suggested that age of target population and the indigenous causes attributing to ambient air pollutants level are important factors to be considered when studying the effect of air pollutants on respiratory health and added supporting evidence to the acute effect of air pollutants including PM_10_, PM_2.5_, NO_2_ and O_3_ on children with asthma.
